# Early left ventricular diastolic function and disease severity in congenital diaphragmatic hernia

**DOI:** 10.1038/s41390-025-04560-6

**Published:** 2025-11-12

**Authors:** Giulia Vanina Cantone, Abhijit Bhattacharya, David Healy, Dana Levinkopf, Anna Claudia Massolo, Flaminia Pugnaloni, Giovanni Vento, Anne Marie Heuchan, Florian Kipfmueller, Neil Patel

**Affiliations:** 1 Department of Neonatology, Fondazione Policlinico Universitario “A. Gemelli” IRCCS, Università Cattolica del Sacro Cuore, Rome, Italy; 2 Department of Neonatology, Royal Hospital for Children, Glasgow, UK; 3 Neonatal Intensive Care Unit, “Bambino Gesù” Children Hospital - IRCCS, Rome, Italy; 4 Department of Neonatology and Pediatric Intensive Care, University Medical Center Mannheim, University Heidelberg, Mannheim, Germany

## Abstract

**Background:**

Congenital diaphragmatic hernia (CDH) remains a major cause of neonatal mortality and morbidity. Early systolic left ventricular (LV) dysfunction is well described in CDH, while diastolic impairment remains unexplored. This study aimed to investigate the feasibility LV diastolic function assessment and to evaluate its relationship with clinical severity.

**Methods:**

Observational, single-centre cohort study. In newborns with CDH, gestational age >32 weeks, echocardiographic assessment of diastolic function evaluation was performed at < 24 hours of age. Data were compared to normative values and correlated to clinical status and outcomes.

**Results:**

Fifty-two infants were included, gestational age 38.0 (32.1–40.3) weeks. Diastolic function in CDH vs. normative controls were: LV mitral E wave 47 ( ± 14) vs. 56 ( ± 8) cm/sec, *p* < 0.001; LV tissue Doppler early diastolic velocity (e’) 4.1 ( ± 1.3) vs. 6.7 ( ± 1.4) cm/sec, *p* < 0.001; left atrial reservoir strain (LASr) 21.3 ( ± 8.7) vs 33.3 ( ± 4.4)%, *p* < 0.001; and E:e’ ratio 12 ( ± 4.2) vs. 33.3 ( ± 4.4), *p* < 0.001. LASr correlated with pCO_2_ (r^2^ = 0.22, *p* < 0.001) and FiO_2_ (r^2^ = 0.20, *p* < 0.001); was lower in non-survivors (*p* = 0.027) and infants requiring invasive ventilation at 14 days (*p* = 0.016).

**Conclusions:**

Diastolic impairment is frequent in the first 24 hours of life in CDH and may contribute to disease severity.

**Impact:**

- Early cardiac ultrasound assessment is crucial in management of congenital diaphragmatic hernia (CDH). Our current investigation addresses the unanswered question of left ventricular (LV) diastolic function and its contribution to pulmonary hypertension and disease severity in CDH.
- We investigated the feasibility of assessing left ventricular (LV) diastolic function in infants with CDH in the first 24 hours of life. Using a panel of echocardiographic measures, including the innovative technique of left atrial strain analysis, assessment of diastolic function was feasible in this cohort.
- Reduced LV diastolic echocardiographic parameters were observed in >70% of the CDH cohort, compared to normative reference data.
- LV diastolic function parameters correlated with echocardiographic estimates of pulmonary artery pressure and measures of global LV systolic function, indicating potential mechanistic interactions and re-consideration of established therapies.
- In CDH cases, early LV diastolic function correlated with clinical parameters and outcomes. Specifically, left atrial strain (LASr) was lower in non-survivors and patients who remained ventilated at 14 days of age. LASr was inversely correlated with maximum arterial PaCO_2_ and maximum inspired fraction of oxygen (FiO_2_) values in the first 24 hours of life, and positively correlated with maximum post-ductal oxygen saturation of haemoglobin (SpO_2_).

## Introduction

Congenital diaphragmatic hernia (CDH) is characterized by a defect in the fetal diaphragm of variable size, which permits intrathoracic herniation of abdominal viscera and is associated with aberrant pulmonary and cardiovascular development.^1^ CDH occurs in 1:2500–5000 live births, and despite ongoing advances in the optimisation of prenatal and postnatal care, mortality remains up to 50% in the most severe cases.

Cardiac dysfunction is an important determinant of pathophysiology and disease severity in CDH.^2^ Early postnatal left ventricular (LV) dysfunction has been observed in over 60% of cases, in isolation or in combination with right ventricular (RV) dysfunction, and is independently associated with adverse outcome in CDH.^3^

Whilst systolic LV dysfunction is well described in CDH, the extent of diastolic LV dysfunction remains less certain. Diastolic function relies on a series of complex interrelated events, including active ventricular relaxation, elastic recoil, and chamber stiffness.^4^

In CDH diastolic function might conceivably be affected by altered myocardial development or extrinsic compression by herniating abdominal contents. Early diastolic dysfunction has been demonstrated in the RV in infants with CDH and is associated with adverse early clinical outcomes.^5^ However, LV diastolic function has not been extensively investigated and could be another important contributor to the development of post-capillary pulmonary hypertension (PH) in this condition.

Cardiac conductance catheterization is the gold standard for assessment of diastolic function, left ventricular filling pressure (LVFP), left atrial pressure (LAP), and pulmonary artery pressure (PAP); however, this invasive technique is neither practical nor safe in the neonatal setting. Clinical echocardiographic assessment of LV diastolic function and LVFP has evolved in the recent decade, driven by emerging recognition of diastolic dysfunction in adult heart disease.^6^ A combination of parameters, including Doppler measures of diastolic blood flow, myocardial velocities, and left atrial deformation (strain) is recommended in adult clinical practice.^7,8^ These techniques may be useful in infants with CDH to investigate LV diastolic function, LVFP, and their contribution to disease pathophysiology.

## Aim

The aim of this study was to assess the feasibility of evaluating left ventricular diastolic function in infants with CDH in the first 24 hours of life and to evaluate its relationship with clinical disease severity.

## Methods

A retrospective observational study was conducted at the Royal Hospital for Children, Glasgow, UK. The study included all neonatal patients diagnosed pre- or postnatally with CDH, with a gestational age at birth of >32 weeks and admitted between January 2017 to November 2024, who received an echocardiographic assessment within the first 24 hours of life. Eligible cases were identified from a review of the electronic health record. Pre-operative echocardiograms, if present, were analysed as well. Cases with significant, life-limiting associated anomalies, including major chromosomal variants, were excluded. Research ethics and institutional data access approvals were obtained.

### Demographic, CDH characteristics, and outcome data

Demographic data collected were sex, gestational age at birth, mode and location of delivery, and birth weight. CDH characteristics collected were prenatal diagnosis, fetal observed-to-expected lung head ratio [o:e LHR], side, size of defect based on CDH Study Group (CDHSG) stages A–D, and liver position. Outcomes of interest were survival to neonatal discharge, use of extra-corporeal membrane oxygenation (ECMO), age at surgery, timing of CDH repair ( > 7 days of life), and ongoing requirement for invasive ventilation at 14 days of life (DoL).

### Echocardiographic parameters

Echocardiographic assessment was performed by two experienced clinicians trained in functional echocardiography (N.P. and A.B.) using a Vivid S70N ultrasound machine with a 6-MHz transducer (GE Medical Systems). Data were collected according to a comprehensive standardised protocol, based on current international guidelines.^9^ Data were analyzed offline by a single investigator (G.V.C.), blinded to patient therapies and outcome, using a dedicated workstation (EchoPAC version 206; GE Medical Systems). In patients who received ECMO, echocardiographic data were collected prior to commencement of ECMO.

Parameters selected to assess diastolic function were based on modified European Association of Cardiovascular Imaging (EACVI) and American Society of Echocardiography guidance, specifically pulsed-wave mitral valve inflow velocities, pulsed-wave tissue Doppler velocities in the basal left ventricular free wall, and left atrial reservoir strain.^6^

**Fig. 1:**
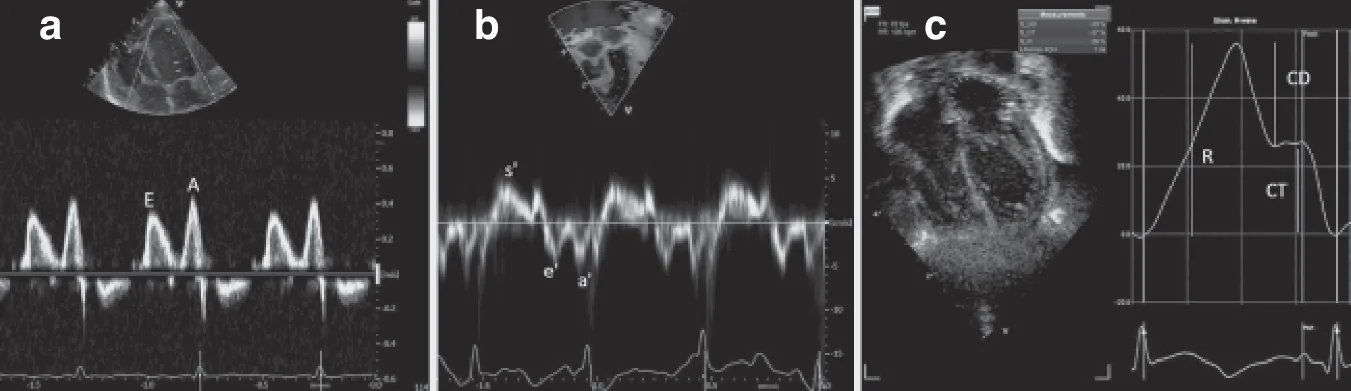
Echocardiographic measures of LV diastolic function and LV filling pressure. **a** Mitral inflow pulsed wave Doppler: E wave represents early diastolic filling phase, A wave represents filling due to later atrial contraction phase. **b** Pulsed Wave Tissue Doppler Imaging myocardial velocities at mitral annulus: s’, systolic ejection velocity; e’, early diastolic velocity; a’, late diastolic velocity. **c** Left atrial strain from measurement: R reservoir phase, CD conduit phase, CT contraction phase.

Pulsed-wave mitral valve inflow velocities were obtained from an apical four-chamber view, using color Doppler the sample gate was first positioned in the peak laminar flow just beyond the mitral valve leaflet tips, with care to minimize the angle of insonation ( < 20^°^). Peak early (E) and late (A) velocities were measured and expressed as the E/A ratio (Fig. 1a). Pulsed-wave tissue Doppler imaging (TDI) was used to obtain peak longitudinal myocardial velocities in systole (LV s’), early (LV e’) and late (LA a’) diastole, in the basal left ventricle, also from a four chamber view (Fig. 1b).^10^ Combining these parameters, the E:e’ ratio was calculated as a composite estimate of left atrial pressure.^11^ This parameter is based on the supposition that E inflow velocities will increase, or “pseudo-normalise” as left atrial pressure increases, whilst e’ tissue velocities reduce with impaired early diastolic relaxation.

Left atrial strain (LAS), assessed using speckle-tracking echocardiography, is an established parameter for the assessment of LA function and LVFP in adults.^12^ This measurement describes the relative change in atrial myocardial length (strain) throughout the cardiac cycle. Three components of atrial deformation are obtained:

- LA reservoir strain (LASr): Corresponding to the maximum relaxation of the atrial wall (positive % value) during ventricular contraction when the LA acts as reservoir for incoming pulmonary venous blood.
- LA conduit strain (LAScd): corresponding to passive atrial shortening after mitral valve opening (negative % value) during early ventricular relaxation as blood flows passively into the ventricle and the LA acts as a conduit between the pulmonary veins and LV,
- LA contraction strain (LASct): corresponding to LA contraction and shortening at the end diastole (negative % value).

LASr has been shown to correlate with pulmonary capillary wedge pressure (PCWP) and be a sensitive and specific measure of LV diastolic dysfunction in adult disease, leading to its inclusion into 2022 EACVI consensus as an additive parameter for the assessment of LVFP.^13,14^

The feasibility of LAS assessment has also been demonstrated in newborn infants, with good inter-observer reproducibility.^12^ As described in these previous studies, LASr was obtained from an apical four-chamber view (Fig. 1c). The region of interest (ROI) was adjusted in size and shape, tracking the atrial endocardial contour with extrapolation across the pulmonary veins and LA appendage. Studies in adult populations have highlighted that endocardial tracking eliminates inter-vendor differences in LASr.^15^ Automated tracking was visually compared to the motion of the underlying atrial wall and accepted only if considered accurate. QRS onset was selected as the reference point and ventricular end diastole as the time reference for the zero-baseline. Following automatic generation of the atrial strain curve, the onset of atrial contraction was designated as the point where the plateau of the curve turns sharply into a downslope, after the beginning of the P-wave in the ECG, as previously described.^16^

Left and right ventricular systolic function were assessed by post-acquisition speckle-tracking (STE) analysis of longitudinal systolic strain (LS) and LV peak systolic strain rate (LV PSR) obtained from the 4-chamber apical view, with optimised frame rate, as previously described.^17^ Systolic function was assessed using LV LS, an effective non-invasive global measure of longitudinal function.^18^ Left ventricular output (LVO) was calculated from Doppler assessment of aortic flow obtained from an apical view, and aortic valve diameter from a parasternal long axis view as previously described.^17^

Pulmonary artery pressure (PAP) was assessed by Doppler assessment of peak tricuspid regurgitation velocity (TRV) and shunting pattern through the patent ductus arteriosus (PDA). Velocity time integers of the right-to-left and left-to-right components of PDA flow were measured by manual tracing and expressed as a ratio.^19^ Pulmonary vascular resistance (PVR) was also assessed by measurement of the pulmonary artery acceleration time: RV ejection time ratio (PAAT:RVET), obtained from the Doppler waveform across the pulmonary valve.

### Clinical management and data collection

All CDH cases were managed according to an established national management protocol, with additional consistent institutional practices. These included intubation and commencement of conventional ventilation in the delivery room with early progression to high-frequency oscillation ventilation (HFOV) in cases of respiratory failure. Early echocardiographic evaluation was performed to assess ventricular function and pulmonary hypertension and to guide cardio-respiratory therapy, including selection of inotropic and pulmonary vasodilator therapies, timing of surgical repair, and use of extracorporeal membrane oxygenation (ECMO).

For all infants, the following parameters were collected during the first 24 hours of life: maximum arterial carbon dioxide and oxygen partial pressures (PaCO_2_ and PaO_2_), highest pre-ductal oxygen saturation (SpO_2_), and maximum FiO_2_.

### Statistical analysis

Echocardiographic parameters were compared between CDH cases and previously reported normative reference data from healthy newborns in the first 24 h of life, grouped by gestational age.^12,20–25^ Patients were divided into ≥ 37 weeks gestational age (GA) or <37 weeks GA groups for statistical analysis. The parameters collected were defined as reduced using cut-off values ± 1 SD from existing normative neonatal data as previously described.^20^ Cut-off values in ≥ 37 weeks GA were: LASr < 28.9%, LV e’<5.3 cm/sec, E/e’ >10.0, LV E/A > 1.2, and in cases of <37 weeks GA were LASr <24.9% LV e’<4.3 cm/sec, E/e’ >13.13 and LV E/A > 1.19.

Correlation was assessed between LV diastolic function parameters and echocardiographic measures of LV and RV systolic function, PAP, and PVR. Correlation was also assessed between LV diastolic function parameters and maximums of PaCO_2_, PaO_2_, SpO_2_, and FiO_2_, within the first 24 h of life after delivery room stabilisation.

Relationships between diastolic function, demographic data, CDH Study Group Stage (A-D), and fetal lung volume (o:e LHR) data were assessed using correlation and subgroup analysis.

LV diastolic function parameters were compared between survivors vs. non-survivors, ECMO vs. no-ECMO, and cases extubated vs. remaining intubated at 14 days of life.

LV diastolic function parameters were compared between early ( < 24 hour) echocardiograms and those performed <24 hours before surgical CDH repair (pre-operative assessment), to evaluate longitudinal changes in diastolic function during the transitional period.

Normalcy of data was assessed using D’Agostino & Pearson test. Welch’s t-testing was used for comparison of continuous variables, Pearson’s χ2 test for categorical variables, and linear regression for correlation analysis. A *p* < 0.05 was considered significant. Analyses were performed using GraphPad Prism 10.4.1 (627).

## Results

Fifty-three patients met the inclusion criteria. One patient was excluded from further analysis due to the presence of severe congenital heart disease (hypoplastic left heart syndrome), subsequent analysis was performed on the remaining fifty-two cases. Demographic data are summarised in Table 1. Median (range) birth weight was 3.00 (1.14–4.10) kg, median (range) gestational age was 38.0 (32.1–40.3) weeks. Forty (77%) of cases had a GA ≥ 37 weeks. CDH was left-sided in 46 (88%) of cases and right-sided in 6 cases (12%). Prenatal diagnosis of CDH was made in 47 cases (90%), in whom the median (range) o:e LHR ratio at prenatal ultrasound was 38% (22 - 81%).

**Table 1:** Demographic data, CDH characteristics, and outcome data.

Demographic data	
**Number of cases**, n	52
**Gestational Age**, weeks, median (range)	38.0 (32.1–40.3)
**Sex, male/ female**, n (%)	32 (62) / 20 (38)
**Birth Weight**, (kg) median (range)	3.00 (1.14–4.10)
**CDH characteristics**	
**Prenatal Diagnosis**, n (%)	47 (90)
**Left Sided**, n (%)	46 (88)
**CDHSG stage A, B, C, D**, n (% of all repaired cases)	A: 5 (10); B: 14 (30); C: 21 (45); D: 7(15)
**Liver in chest**, n (% of all repaired cases)	28 (60)
**Outcomes**	
**Surgical repair of CDH**, n (%)	47 (87)
**Patch repair**, n (%)	35 (74)
**ECMO**, n (%)	12 (23)
**Survival**, n (%)	42 (81)
**Duration of invasive ventilation**^a^, days, median (range)	14 (3–77)
**Hours of life at first echo**, hours, median (range)	4.5 (0.5–24)
**Heart rate at echo**, beats per minute, median (range)	146 (112–193)

*CDHSG* congenital diaphragmatic hernia study group,

*ECMO* Extra corporeal membrane oxygenation.

^a^in survivors.

Forty-seven (90%) cases received CDH repair of whom 35 (75%) required a patch, 28 cases (60%) had liver in their chest. Of repaired cases, 60% had a larger type C or D defect. Twelve cases (23%) received ECMO, overall survival was 42 (81%).

During the first 24 hours of life, 30 cases (58%) received conventional mechanical ventilation only, the remaining 22 (42%) received HFOV. During the subsequent neonatal period, intravenous epinephrine was administered in 31 cases (60%), milrinone in 27 (52%) cases, dopamine in 15 (29%) cases, and norepinephrine and vasopressin in 1 case each (2%–2%). Inhaled nitric oxide was administered in 25 cases (48%). Intravenous prostaglandin E1 was utilised in 2 patients (5%), and one patient received intravenous sildenafil (2%).

In the included 52 subjects, initial echocardiograms were performed at median (range) 5 (0.5-24) hours of life. LASr was obtained in all 52 cases (100%), MV E/A in 42 cases (81%), LV e’ in 42 patients (81%) and E/e’ ratio in 40 patients (77%). Missing data and insufficient image quality prevented the collection of LV e’ and E/e’ data in the remaining cases.

In the GA > 37 weeks group, a patent ductus was present in forty-seven cases (90%), flow in these was bidirectional in twenty-one cases (40%), right-to-left in sixteen cases (31%), and left-to-right in five cases (10%). Interatrial shunt direction, when available and present, was bidirectional in seven (32%), left-to-right in twelve (54%), and right-to-left in three (14%).

**Fig. 2:**
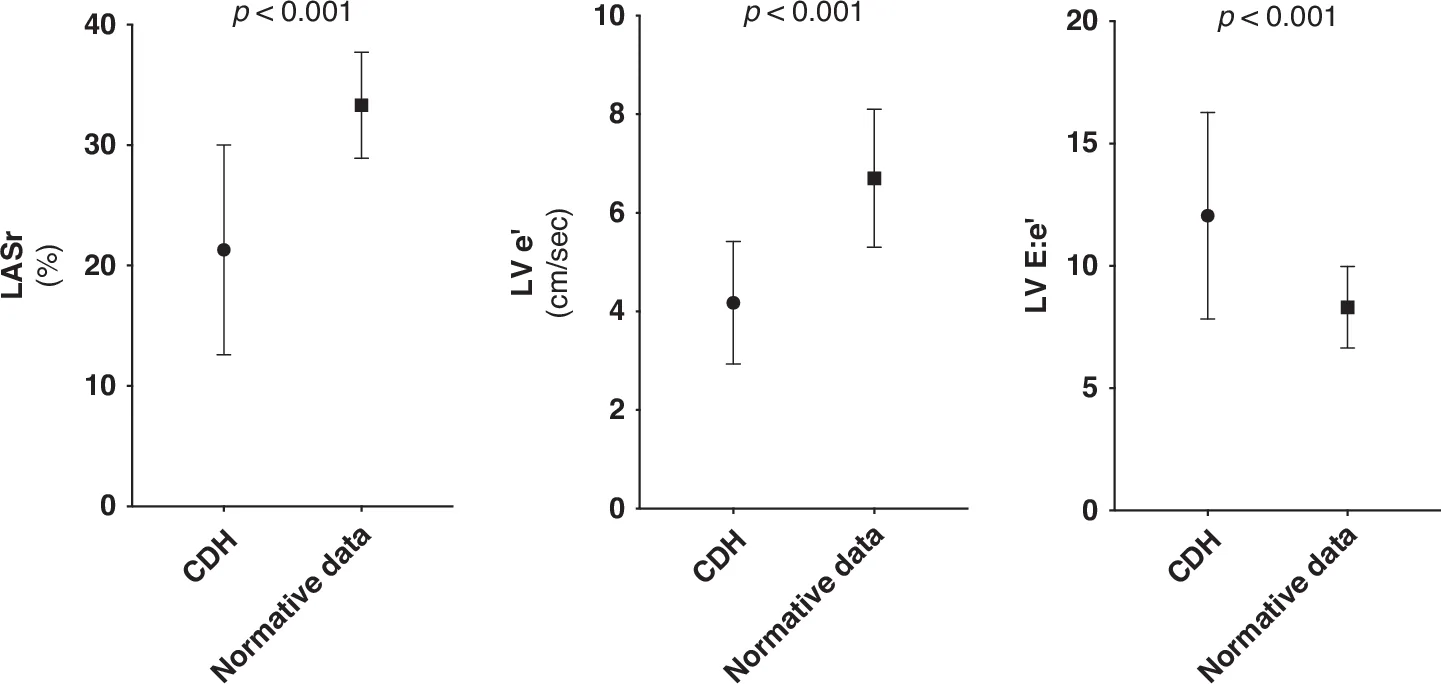
Measures of left ventricular diastolic function in infants with CDH and normative data in the first 24 h of life. LASr left atrial reservoir strain, LV e’ left ventricular early diastolic myocardial velocity, LV E:e’ ratio of mitral valve early diastolic filling velocity E and LV e’.

LV diastolic parameters were reduced compared to existing normative data in the ≥37 weeks GA group, Table 2: LASr 21.3 ( ± 8.7) vs. 33.3 ( ± 4.4), LV e’ 4.1 ( ± 1.3) vs. 6.7 ( ± 1.4), E/A 1.0 ( ± 0.3) vs 1.1 ( ± 0.1), *p* = 0.028, whilst E/e’ was increased in CDH, 12 ( ± 4.2) vs 8.3 ( ± 1.4), all *p* < 0.001, Fig. 2. In the <37 weeks GA group, LV e’ and LASr were reduced compared to normative reference values, respectively LASr 23.5 ( ± 12) vs. 33.3 ( ± 8.4), *p* < 0.001, and LV e’ 4.3 ( ± 1.2) vs. 6.2 ( ± 1.0), *p* = 0.022. Utilising a priori cut-offs, LASr was reduced in 73% of all CDH cases, MV E/A ratio in 26% of cases, and LV e’ in 65% of cases, with an associated increase in E/e’ in 48% of cases.

**Table 2:** Measures of left ventricular diastolic function in CDH cases and comparison with normative data.

	All CDH (*n* = 52)	CDH > 37 w (*n* = 40)	Normative data Term	p	CDH < 37 w (*n* = 12)	Normative data Preterm	p
**Peak Mitral E** cm/sec, mean (SD)	45 ( ± 13)	47 ( ± 14)	56 ( ± 8)	**<0.001**	40 ( ± 8)	56 ( ± 9)	**<0.001**
**Peak Mitral A** cm/sec, mean (SD)	47 ( ± 12)	47 ( ± 11)	50 ( ± 8)	**0.03**	48 ( ± 16)	59 ( ± 15)	**0.04**
**E/A ratio** mean (SD)	1.0 ( ± 0.3)	1.0 ( ± 0.3)	1.1 ( ± 0.1)	**0.03**	0.9 ( ± 0.4)	0.99 ( ± 0.2)	0.59
**LV e’** cm/sec, mean (SD)	4.2 ( ± 1.2)	4.1 ( ± 1.3)	6.7 ( ± 1.4)	**<0.001**	4.3 ( ± 1.2)	6.2 ( ± 1.0)	**0.02**
**E/e’ ratio** mean (SD)	11.6 ( ± 4.7)	12 ( ± 4.2)	8.3 ( ± 1.4)	**<0.001**	10.2 ( ± 6.1)	10.3 ( ± 2.8)	0.96
**LASr** %, mean (SD)	21.8 ( ± 9.5)	21.3 ( ± 8.7)	33.3 ( ± 4.4)	**<0.001**	23.5 ( ± 12)	33.3 ( ± 8.4)	**<0.001**
**LV LS** %, mean (SD)	−13.6 ( ± 5.3)	−12.9 ( ± 5.2)	−21.7 ( ± 1.9)	**<0.001**	−16.5 ( ± 4.9)	−12.2 ( ± 2.8)	**0.011**
**RV LS** %, mean (SD)	−11.8 ( ± 5.4)	−11.1( ± 6.4)	−21.2 ( ± 5.3)	**<0.001**	12.5 ( ± 4.6)	−22.8 ( ± 4.8)	**<0.001**

*E* peak early mitral diastolic inflow velocity, *A* peak late mitral diastolic inflow velocity, *E/A* ratio of peak mitral E and A diastolic velocities, *LV e’* left ventricular early diastolic myocardial velocity, *LV E:e’* ratio of mitral valve early diastolic filling velocity E and LV e’, *LASr* left atrial reservoir strain, *LV LS* Left ventricular longitudinal strain, *RV LS* right ventricular longitudinal strain.

*p* values <0.05 statistically significant has been written in bold to highlight the result.

LV LS in the ≥37 weeks GA group was −12.90 ( ± 5.3)% vs. reference values in term newborns on day 1 of life −21.7 ( ± 1.9)% p < 0.001. RV LS in the ≥37 weeks GA group was – 11.1 ( ± 6.4)% vs. reference values −21.2 ( ± 5.3)% *p* < 0.001.

**Fig. 3:**
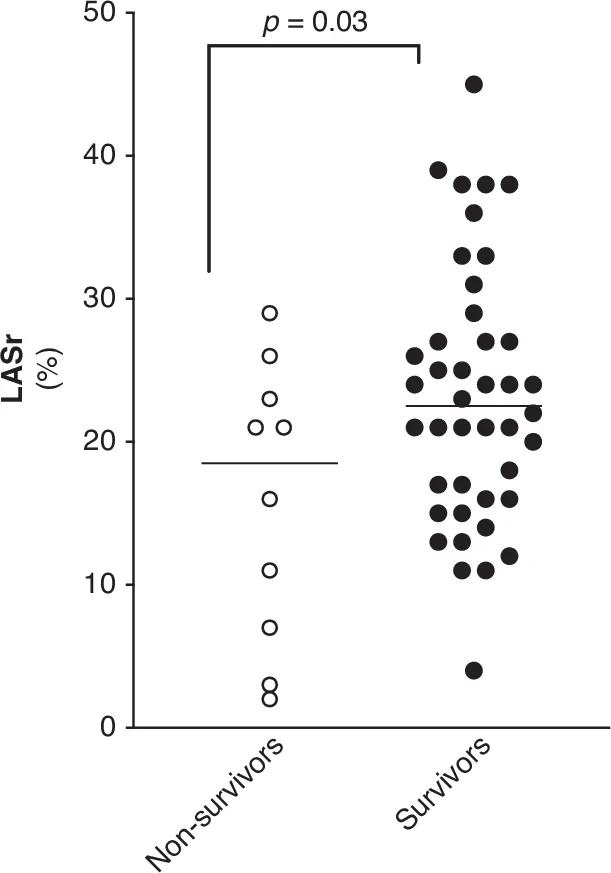
Left atrial reservoir strain and survival in CDH cases in the first 24 h of life. LASr left atrial reservoir strain.

LASr was lower in cases with CDHSG stage AB vs. CD (p = 0.034) and in CDH cases with a fetal o:e LHR < 40% (*p* = 0.026), Table 3. LASr was lower in non-surviving cases (*p* = 0.034), Fig. 3, those who remained invasively ventilated at > 14 DoL (*p* = 0.011), and those who received surgical repair at or after 7 DoL (*p* = 0.029). No statistical difference was observed in LASr in infants who received ECMO vs. non-ECMO cases (*p* = 0.091), Table 3.

**Table 3:** LV diastolic function parameters at < 24 h of age, CDH characteristics and outcomes.

CDH characteristic or outcome		LASr		LV e’		LV E/e’	
		Mean (SD)	p	Mean (SD)	p	Mean (SD)	p
**CDHSG Stage**	**AB**, *n* = 19	26.2 ( ± 9.1)	**0.03**	4.1 ( ± 0.7)	0.98	12.4 ( ± 3.3)	0.26
	**CD**, *n* = 24	20.6 ( ± 8.0)		4.1 ( ± 1.2)		10.9 ( ± 4.5)	
**o:e LHR**	**o:e LHR** < **40**, *n* = 23	17.8 ( ± 7.5)	**0.03**	4.4 ( ± 1.4)	0.35	10.5 ( ± 4.9)	0.11
	**o:e LHR** ≥ **40**, *n* = 21	24.1 ( ± 10.3)		4.0 ( ± 1.1)		13.1 ( ± 4.6)	
**Survival**	**Survivors**, *n* = 42	23.2 ( ± 9.0)	**0.03**	4.1 ( ± 1.1)	0.16	11.9 ( ± 4.0)	0.47
	**Non-surv**., *n* = 10	15.9 ( ± 9.7)		4.8 ( ± 1.8)		10.2 ( ± 8.6)	
**ECMO**	**ECMO**, *n* = 12	17.9 ( ± 6.4)	0.09	4.7 ( ± 1.3)	0.16	8.0 ( ± 2.6)	**0.007**
	**Non ECMO**, *n* = 40	23.5 ( ± 10.2)		4.1 ( ± 1.2)		12.4 ( ± 4.6)	
**Invasive Ventilation** ≥ **14 DoL**	**Yes**, *n* = 22	19.9 ( ± 7.7)	**0.01**	4.0 ( ± 1.1)	0.50	11.4 ( ± 4.6)	0.45
	**No**, *n* = 20	26.9 ( ± 9.2)		4.2 ( ± 1.0)		12.4 ( ± 3.2)	
**Repair** ≥ **7 DoL**	**Yes**, *n* = 22	19.6 ( ± 7.5)	**0.03**	4.1 ( ± 1.4)	0.92	11.2 ( ± 4.2)	0.73
	**No**, *n* = 24	24.9 ( ± 8.3)		4.1 ( ± 0.8)		11.7 ( ± 4.2)	

*CDHSG stage* Congenital Diaphragmatic Hernia Study Group defect stage, *o:e* *LHR* observed:expected lung to head ratio, *ECMO* extracorporeal oxygenation membrane oxygenation, *DoL* days of life, *LV e*’ left ventricular early diastolic myocardial velocity, *LV E:e*’ ratio of mitral valve early diastolic filling velocity E and LV e’; *LASr* left atrial reservoir strain.

*p* values <0.05 statistically significant has been written in bold to highlight the result.

**Fig. 4:**
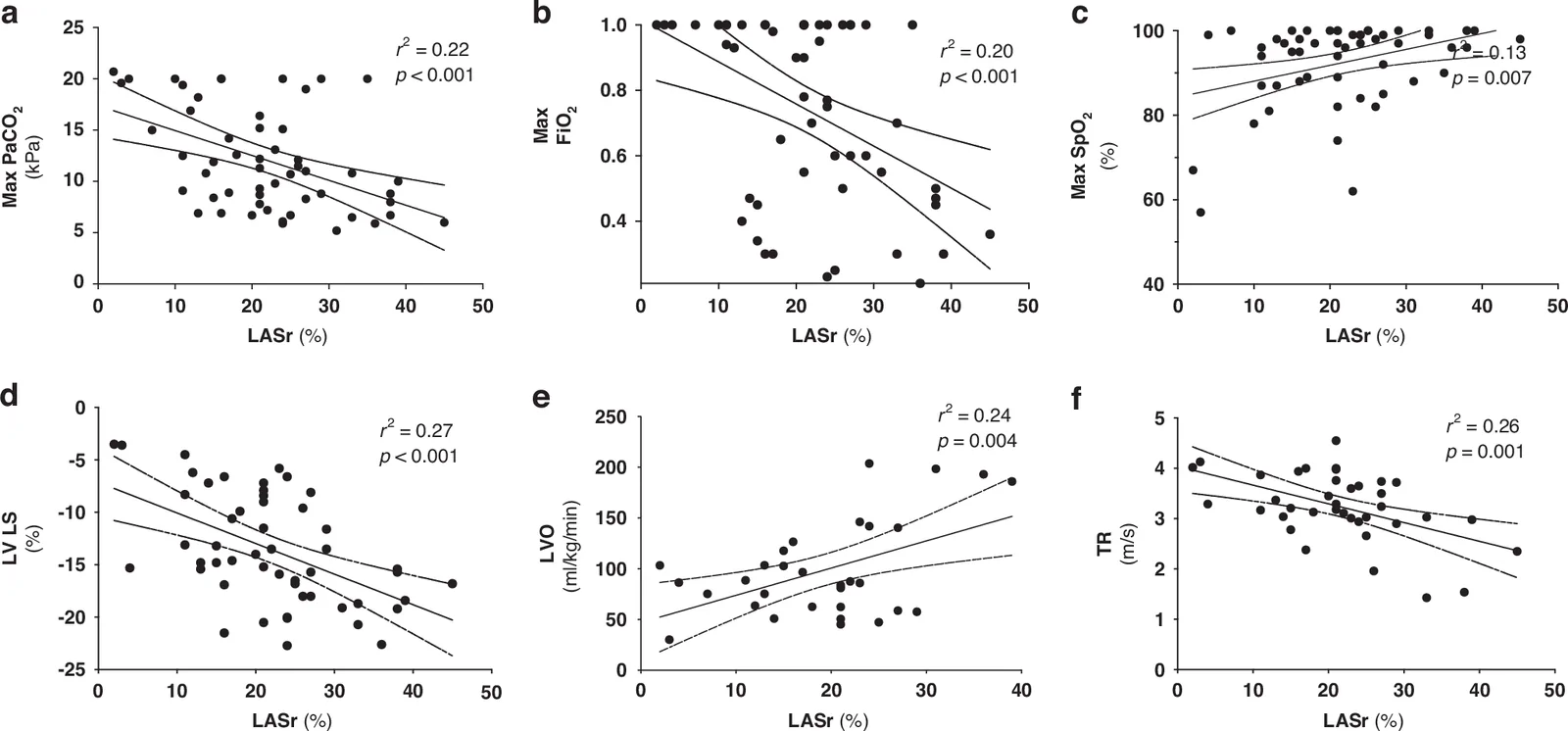
Correlation of left atrial reservoir strain with clinical and echocardiographic parameters. **a** Left atrial reservoir strain (LASr) and maximum arterial partial pressure of carbon dioxide (max PaCO_2_). **b** LASr and maximum inspired oxygen fraction (max FiO_2_), **c** LASr and maximum arterial oxygen saturation (max SpO_2_), **d** LASr and left ventricular longitudinal systolic strain (LV LS), **e** LASr and left ventricular output (LVO), **f** LASr and maximum velocity of tricuspid regurgitation (TR).

LASr demonstrated inverse correlation with maximum PaCO_2_ (r^2^ = 0.22, *p* < 0.001), maximum FiO_2_ (r^2^ = 0.20, *p* < 0.001) and positive correlation with maximum SpO_2_ (r^2^ = 0.13, *p* = 0.007), Fig. 4. LVLS inversely correlated with maximum PaCO_2_ and maximum post-ductal SpO_2_ respectively (r^2^ = 0.32, *p* < 0.001 and r^2^ = 0.40, *p* < 0.001. No other correlations were observed between measures of diastolic function and clinical measures of gas exchange.

LASr was correlated with measures of LV systolic function: LV LS (r^2^ = 0.27, *p* < 0.001), LV PSR (r^2^ = 0.68, *p* < 0.001), and with LVO (r^2^ = 0.24, *p* = 0.005). LV e’ correlated with LVs’ (r^2^ = 0.34, *p* < 0.001) and Right Ventricular (RV) LS (r^2^ = 0.13, p = 0.021). LV E:e’ correlated with LV s’ (r^2^ = 0.18, *p* = 0.008).

Maximal TR velocity correlated with LASr (r^2^ = 0.26, *p* = 0.001). PAAT:RVET correlated with both LV e’ (r^2^ = 0.23, *p* = 0.003) and LV E: e’ (r^2^ = 0.14, *p* = 0.029). No other correlations were observed between LV diastolic function and pulmonary hypertension or RV function parameters.

A follow-up pre-operative scan was available in 27 cases, performed at a median of 7 (range 2–14) days of life. LV E, LV e’, E/A ratio, LASr, LV LS, and RV LS all increased between <24 h and pre-operative scans (all *p* < 0.05), Supplementary Table S1.

## Discussion

This preliminary study demonstrated the feasibility of echocardiographic assessment of left ventricular diastolic function in infants with CDH in the first 24 hours life and pre-operative period. Reduced early left ventricular diastolic function was observed in >70% of cases and was associated with clinical status and outcomes including survival. These findings provide new insights into pathophysiological mechanisms of cardio-respiratory dysfunction in CDH during the neonatal transition and may be relevant to improving management strategies.

Direct assessment of diastolic function by conductance catheter is neither safe nor feasible in newborn infants. Instead, we utilised echocardiographic parameters, including left atrial strain, recommended in adult clinical practice.^6,11,14^ The requirement for multiple parameters highlights the complex nature of diastolic function and the fact that no single measure can be considered a gold standard.^4^

In preterm infants De Waal et al have previously demonstrated the feasibility of using E/A, LV e’, and E:e’, combined with LA volumes for assessment of diastolic function.^20^ Left atrial strain assessment has additionally been demonstrated, and normative data reported, in preterm and term infants by Ficial et al.^12^ In newborn infants the interpretation of these parameters is complicated by the need to consider the effects of atrial or ductal shunts, dynamic changes in pulmonary and systemic vascular resistances, and transitional changes in myocardial structure and function.^26^

The present study confirms the feasibility of LV diastolic function assessment in CDH. Abnormalities in LASr were most frequent, followed by LV e’ and LV E: e’ ratio, suggesting that these may be more sensitive clinical measures and confirming similar findings in preterm populations.^27^ The feasibility of LASr assessment in CDH was also recently reported in a retrospective study by Tyden et al., who observed abnormal LASr in 77% of cases in the first 72 hours of life in a similar-sized cohort.^28^ In that study, a higher cut-off of LASr <33% was used to define abnormal LASr, which, if applied in our cohort, would have included all cases. Of note, when comparing these studies, the current investigation was performed earlier in the first 24 hours of life and may have included a greater proportion of more severe CDH cases with larger diaphragmatic defects, higher ECMO use, and lower overall survival. We also used a different ultrasound device and post-processing software.

Diastolic function comprises an early phase (E) of active myocardial relaxation combined with elastic recoil of the ventricle, releasing energy stored in systole. Later ventricular filling (A) is due to atrial contraction. Ventricular stiffness provides the resistance to filling.^29^ The observed reductions in myocardial LV e’ indicate impaired myocardial relaxation in the CDH cohort. The increase in E:e’ and reduction in LA reservoir strain meanwhile, suggest a concomitant increase in LAP indicative of increased ventricular stiffness (reduced compliance). In adults, elevated E velocity is used as a screening measure of LV diastolic dysfunction, based on a presumption of increased ventricular stiffness leading to a restrictive filling pattern. In CDH infants, however, we observed a net *reduction* in E velocity. This likely reflects the co-existent opposing effects of impaired relaxation (which reduces E velocity) and increased chamber stiffness (which increases E velocity, and E:e’), and highlights the need for caution when applying adult parameters to neonatal settings.

An additional consideration in neonates is the presence of atrial and ductal shunts in the transitional circulation. In preterm infants, volume loading due to sizeable left-to-right ductal shunts is associated with reduced LASr strain and increased E velocity.^26^ Ductal shunts in the current cohort were predominantly bidirectional or right-to-left.

A number of underlying pathophysiological mechanisms may contribute to impaired LV diastolic function in CDH. First, developmental hypoplasia of the left ventricle is well described in CDH, and may be accompanied by abnormalities of cell structure, perfusion, and metabolic function.^2,30–32^ Second, RV dysfunction and dilation, in response to increased afterload, may impact LV diastolic function through mechanisms of ventricular interdependence, including shared muscle fibres and septal displacement.^33^ Third, external compression of the LV by herniating abdominal contents. Fourth, impaired systolic function may lead to combined “systo-diastolic dysfunction” through mechanisms including reduced elastic recoil, impaired atrial filling during systole, and dyssynchrony.^17,34,35^ In support of this mechanism we observed impaired systolic function of both left and right ventricles, correlation of LV systolic and diastolic function parameters, and simultaneous improvement in these from <24 h to pre-operative timepoints. Fifth, as in adult heart failure, increased systemic vascular resistance may play a role in diastolic dysfunction.^36^ In neonates, elevated SVR is a specific finding in the transition from fetal to postnatal life.

We observed lower LASr in non-survivors and in cases requiring intubation beyond two weeks of age. Tyden et al. previously observed a relationship between LASr in CDH and length of neonatal stay, though not survival or ECMO use. These differences in findings may be due to variation in management between cohorts and earlier timing of LASr assessment in our study. Nevertheless, taken together, these observations point to the potential adverse clinical impact of left heart diastolic dysfunction in CDH, which may be mediated by a number of potential mechanisms.

PH in CDH has predominantly been attributed to a *pre-capillary* increase in pulmonary arterial vascular resistance.^37^ However, our findings provide evidence for alternative mechanisms of *post-capillary* PH in early CDH, due to elevated LAP, LVFP, and pulmonary venous hypertension secondary to LV diastolic dysfunction.^38^ In support of these mechanisms, we observed correlations between LV diastolic function and measures of pulmonary artery pressure (maximal TR velocity and PAAT:RVET ratio). The absence of a stronger correlation, or correlation with all measures of PAP or PVR, highlights the challenge of accurate, non-invasive PAP and PVR assessment in newborns, and that these are elevated in all infants in the first 24 hours of life as a component of the transitional circulation.^36^

Reduced diastolic function and associated elevated pulmonary venous pressure may also affect clinical status by causing pulmonary venous congestion and pulmonary oedema, in turn impairing lung mechanics and gas exchange.^37^ Consistent with this mechanism of heart-lung interaction, we observed correlation between diastolic function, PaCO_2_, and oxygenation in our cohort. These correlations were not absolute, likely reflecting the multi-factorial pulmonary and hemodynamic factors influencing gas exchange in CDH, of which diastolic function may be one component.

Our findings may have important therapeutic implications in CDH. In this setting of LV diastolic dysfunction and pulmonary venous hypertension, the use of pulmonary vasodilators is likely to be ineffective or indeed detrimental, which may explain the recently reported adverse association of inhaled nitric oxide with outcome in CDH.^39,40^ Furthermore, fluid boluses are likely to exacerbate pulmonary congestion and the temptation to increase ventilation pressures to treat hypercarbia and hypoxia risks lung injury without addressing the underlying haemodynamic mechanism.

Based on our findings, we instead advocate for a treatment strategy that incorporates routine echocardiographic assessment and direct inotropic and lusitropic (diastolic) support of myocardial function, avoiding deleterious interventions.^41^ Early CDH repair, within the first 24 h of life, has also been proposed as a means of improving LV diastolic function; however, this may be balanced against the potentially destabilising effects of surgery and the spontaneous improvement in LV systolic and diastolic within the first days of life as observed in the current investigation.^42,43^

### Limitations

We used existing normative data for comparison rather than a study-specific control group. This reflects usual clinical practice and is in alignment with the approach taken in preterm studies.^20^ Inter-vendor differences in ultrasound devices and post-processing software are an important consideration in these comparisons, but are potentially mitigated by our use of endocardial tracking.^15^ The limited cohort size reflects the wider challenge of researching this rare condition. Further investigation in larger multi-centre cohorts is required to confirm our findings. We did not include left atrial volumes or pulmonary vein velocities due to the absence of sufficient data, this is an area for future investigation. Heart rate may also affect ventricular function, tachycardia, in particular, will shorten diastolic time intervals.^44^ Six infants had a heartrate >180 bpm at the time of echocardiographic assessment; however median heart rate was within normal limits. Concomitant cardio-respiratory therapies may have influenced the measured parameters and observed relationships; however, the effects of these were minimised by use of an established treatment protocol and early echocardiographic assessment ( <24 hours) before initiation of most therapies. Furthermore, this study was not designed to investigate the individual effects of therapy, but rather as a preliminary exploration of the feasibility of diastolic function assessment.

## Conclusion

Impaired diastolic function in the left heart is common in CDH during the early transitional period and may be an important mechanism of post-capillary pulmonary hypertension. Diastolic function is associated with clinical status, including gas exchange and neonatal outcomes. Early echocardiographic assessment may enable detection of this pathophysiological phenotype and guide improved management strategies in the future, including judicious use of pulmonary vasodilators, fluid boluses, and higher pressure ventilation.

## Supplementary information

Supplementary Table S1

## Data Availability

The datasets generated and/or analyzed during the current study are available from the corresponding author on reasonable request and approval from the study team.

## References

[1] Zani, A. et al. Congenital diaphragmatic hernia. *Nat. Rev. Dis. Prim.* **8**, 37 (2022). 10.1038/s41572-022-00362-w 35650272

[2] Patel, N., Massolo, A. C., Kraemer, U. S. & Kipfmueller, F. The heart in congenital diaphragmatic hernia: Knowns, unknowns, and future priorities. *Front. Pediatr.* **10**, 890422 (2022). 10.3389/fped.2022.890422 PMC9424541 36052357

[3] Patel, N. et al. Ventricular Dysfunction Is a Critical Determinant of Mortality in Congenital Diaphragmatic Hernia. *Am. J. Respir. Crit. Care Med.* **200**, 1522–1530 (2019). 10.1164/rccm.201904-0731OC 31409095

[4] Nishimura, R. A. & Borlaug, B. A. Diastology for the clinician. *J. Cardiol.* **73**, 445–452 (2019). 10.1016/j.jjcc.2019.03.002 30922613

[5] Moenkemeyer, F. & Patel, N. Right Ventricular Diastolic Function Measured by Tissue Doppler Imaging Predicts Early Outcome in Congenital Diaphragmatic Hernia. *Pediatr. Crit. Care Med.* **15**, 49–55 (2014). 10.1097/PCC.0b013e31829b1e7a 23925147

[6] Nagueh, S. F. et al. Recommendations for the Evaluation of Left Ventricular Diastolic Function by Echocardiography: An Update from the American Society of Echocardiography and the European Association of Cardiovascular Imaging. *J. Am. Soc. Echocardiogr.* **29**, 277–314 (2016). 10.1016/j.echo.2016.01.011 27037982

[7] Popescu, B. A., Beladan, C. C., Nagueh, S. F. & Smiseth, O. A. How to assess left ventricular filling pressures by echocardiography in clinical practice. *Eur. Heart J. - Cardiovasc. Imaging* **23**, 1127–1129 (2022). 10.1093/ehjci/jeac123 35762650

[8] Hassan, O. K. A. & Higgins, A. R. The role of multimodality imaging in patients with heart failure with reduced and preserved ejection fraction. *Curr. Opin. Cardiol.* **37**, 285–293 (2022). 10.1097/HCO.0000000000000963 35612940

[9] McNamara, P. J. et al. Guidelines and Recommendations for Targeted Neonatal Echocardiography and Cardiac Point-of-Care Ultrasound in the Neonatal Intensive Care Unit: An Update from the American Society of Echocardiography. *J. Am. Soc. Echocardiogr.* **37**, 171–215 (2024). 10.1016/j.echo.2023.11.016 38309835

[10] Patel, N., Mills, J. F. & Cheung, M. M. H. Assessment of Right Ventricular Function Using Tissue Doppler Imaging in Infants with Pulmonary Hypertension. *Neonatology* **96**, 193–199 (2009). 10.1159/000215585 19407463

[11] Robinson, S. et al. The assessment of left ventricular diastolic function: guidance and recommendations from the British Society of Echocardiography. *Echo Res. Pract.* **11**, 16 (2024). 10.1186/s44156-024-00051-2 PMC11145885 38825710

[12] Ficial, B. et al. Feasibility, Reproducibility and Reference Ranges of Left Atrial Strain in Preterm and Term Neonates in the First 48 h of Life. *Diagnostics* **12**, 350 (2022). 10.3390/diagnostics12020350 PMC8871374 35204441

[13] Silva, M. R., Sampaio, F., Braga, J., Ribeiro, J. & Fontes-Carvalho, R. Left atrial strain evaluation to assess left ventricle diastolic dysfunction and heart failure with preserved ejection fraction: a guide to clinical practice: Left atrial strain and diastolic function. *Int. J. Cardiovasc. Imaging* **39**, 1083–1096 (2023). 10.1007/s10554-023-02816-y 36826616

[14] Smiseth, O. A. et al. Multimodality imaging in patients with heart failure and preserved ejection fraction: an expert consensus document of the European Association of Cardiovascular Imaging. *Eur. Heart J. - Cardiovasc. Imaging* **23**, e34–e61 (2022). 10.1093/ehjci/jeab154 34729586

[15] Ferkh, A. et al. Inter-vendor comparison of left atrial strain using layer specific strain analysis. *Int. J. Cardiovasc. Imaging* **37**, 1279–1288 (2021). 10.1007/s10554-020-02114-x 33389361

[16] Badano, L. P. et al. Standardization of left atrial, right ventricular, and right atrial deformation imaging using two-dimensional speckle tracking echocardiography: a consensus document of the EACVI/ASE/Industry Task Force to standardize deformation imaging. *Eur. Heart J. - Cardiovasc. Imaging* **19**, 591–600 (2018). 10.1093/ehjci/jey042 29596561

[17] Patel, N. et al. Early Postnatal Ventricular Dysfunction Is Associated with Disease Severity in Patients with Congenital Diaphragmatic Hernia. *J. Pediatr.* **203**, 400–407.e1 (2018). 10.1016/j.jpeds.2018.07.062 30195555

[18] Petoello, E. et al. Global longitudinal strain is an informative index of left ventricular performance in neonates receiving intensive care. *Sci. Rep.* **14**, 8881 (2024). 10.1038/s41598-024-59441-5 PMC11024117 38632330

[19] Bialkowski, A., Moenkemeyer, F. & Patel, N. Intravenous Sildenafil in the Management of Pulmonary Hypertension Associated with Congenital Diaphragmatic Hernia. *Eur. J. Pediatr. Surg.* **25**, 171–176 (2013). 10.1055/s-0033-1357757 24163194

[20] De Waal, K., Costley, N., Phad, N. & Crendal, E. Left Ventricular Diastolic Dysfunction and Diastolic Heart Failure in Preterm Infants. *Pediatr. Cardiol.* **40**, 1709–1715 (2019). 10.1007/s00246-019-02208-x 31598743

[21] Negrine, R. J. S., Chikermane, A., Wright, J. G. C. & Ewer, A. K. Assessment of myocardial function in neonates using tissue Doppler imaging. *Arch. Dis. Child. - Fetal Neonatal Ed.* **97**, F304–F306 (2012). 10.1136/adc.2009.175109 21037287

[22] Jain, A. et al. Left Ventricular Function in Healthy Term Neonates During the Transitional Period. *J. Pediatr.* **182**, 197–203.e2 (2017). 10.1016/j.jpeds.2016.11.003 27908646

[23] Nestaas, E., Schubert, U., de Boode, W. P., El-Khuffash, A. & European Special Interest Group ‘Neonatologist Performed Echocardiography’ (NPE) Tissue Doppler velocity imaging and event timings in neonates: a guide to image acquisition, measurement, interpretation, and reference values. *Pediatr. Res.* **84**, 18–29 (2018). 10.1038/s41390-018-0079-8 PMC6257218 30072806

[24] on behalf of the European Special Interest Group ‘Neonatologist Performed Echocardiography’ (NPE) et al. Deformation imaging and rotational mechanics in neonates: a guide to image acquisition, measurement, interpretation, and reference values. *Pediatr. Res.* **84**, 30–45 (2018). 10.1038/s41390-018-0080-2 PMC6257225 30072804

[25] Jain, A. et al. A Comprehensive Echocardiographic Protocol for Assessing Neonatal Right Ventricular Dimensions and Function in the Transitional Period: Normative Data and Z Scores. *J. Am. Soc. Echocardiogr.* **27**, 1293–1304 (2014). 10.1016/j.echo.2014.08.018 25260435

[26] De Waal, K., Phad, N. & Boyle, A. Left atrium function and deformation in very preterm infants with and without volume load. *Echocardiography* **35**, 1818–1826 (2018). 10.1111/echo.14140 30225864

[27] De Waal, K., Phad, N. & Crendal, E. Echocardiography algorithms to assess high left atrial pressure and grade diastolic function in preterm infants. *Echocardiography* **40**, 1099–1106 (2023). 10.1111/echo.15686 37658834

[28] Tydén, K. Ö., Mesas Burgos, C., Jonsson, B. & Nordenstam, F. Left atrial strain in neonates with congenital diaphragmatic hernia and length of stay in pediatric intensive care unit. *Front. Pediatr.* **12**, 1404350 (2024). 10.3389/fped.2024.1404350 PMC11183789 38895191

[29] Smiseth, O. A. Evaluation of left ventricular diastolic function: state of the art after 35 years with Doppler assessment. *J. Echocardiogr.* **16**, 55–64 (2018). 10.1007/s12574-017-0364-2 PMC5966482 29236226

[30] Nour, A. L. A. et al. The myocardial capillary network is altered in congenital diaphragmatic hernia in the fetal rabbit model. *Braz. J. Med. Biol. Res.* **56**, e12521 (2023). 10.1590/1414-431X2023e12521 PMC10242701 37194833

[31] Zhaorigetu, S., Gupta, V. S., Jin, D. & Harting, M. T. Cardiac energy metabolism may play a fundamental role in congenital diaphragmatic hernia-associated ventricular dysfunction. *J. Mol. Cell. Cardiol.* **157**, 14–16 (2021). 10.1016/j.yjmcc.2021.04.001 33878329

[32] Fraga, M. V. et al. Congenital Diaphragmatic Hernia Patients with Left Heart Hypoplasia and Left Ventricular Dysfunction Have Highest Odds of Mortality. *J. Pediatr.* **271**, 114061 (2024). 10.1016/j.jpeds.2024.114061 PMC11239301 38636784

[33] Altit, G., Lapointe, A., Kipfmueller, F. & Patel, N. Cardiac function in congenital diaphragmatic hernia. *Semin. Pediatr. Surg.* **33**, 151438 (2024). 10.1016/j.sempedsurg.2024.151438 39018716

[34] Altit, G., Bhombal, S., Van Meurs, K. & Tacy, T. A. Ventricular Performance is Associated with Need for Extracorporeal Membrane Oxygenation in Newborns with Congenital Diaphragmatic Hernia. *J. Pediatr.* **191**, 28–34.e1 (2017). 10.1016/j.jpeds.2017.08.060 29037794

[35] Massolo, A. C. et al. Ventricular Dysfunction, Interdependence, and Mechanical Dispersion in Newborn Infants with Congenital Diaphragmatic Hernia. *Neonatology* **116**, 68–75 (2019). 10.1159/000499347 31085915

[36] Sorrentino, M. J. The Evolution from Hypertension to Heart Failure. *Heart Fail. Clin.* **15**, 447–453 (2019). 10.1016/j.hfc.2019.06.005 31472880

[37] Holden, K. I., Rintoul, N. E., McNamara, P. J. & Harting, M. T. Congenital diaphragmatic hernia-associated pulmonary hypertension. *Semin. Pediatr. Surg.* **33**, 151437 (2024). 10.1016/j.sempedsurg.2024.151437 39018718

[38] Bhombal, S. & Patel, N. Diagnosis & management of pulmonary hypertension in congenital diaphragmatic hernia. *Semin. Fetal Neonatal Med.* **27**, 101383 (2022). 10.1016/j.siny.2022.101383 35995665

[39] Noh, C. Y. et al. Early nitric oxide is not associated with improved outcomes in congenital diaphragmatic hernia. *Pediatr. Res.* **93**, 1899–1906 (2023). 10.1038/s41390-023-02491-8 36725908

[40] Loh, E., Stamler, J. S., Hare, J. M., Loscalzo, J. & Colucci, W. S. Cardiovascular effects of inhaled nitric oxide in patients with left ventricular dysfunction. *Circulation* **90**, 2780–2785 (1994). 10.1161/01.cir.90.6.2780 7994821

[41] Surak, A., Mahgoub, L. & Ting, J. Y. Hemodynamic management of congenital diaphragmatic hernia: the role of targeted neonatal echocardiography. *World J. Pediatr. Surg.* **7**, e000790 (2024). 10.1136/wjps-2024-000790 PMC11086387 38737963

[42] Inamura, N. et al. Efficacy of the circulatory management of an antenatally diagnosed congenital diaphragmatic hernia: outcomes of the proposed strategy. *Pediatr. Surg. Int.* **30**, 889–894 (2014). 10.1007/s00383-014-3574-y 25106889

[43] Le, L. S., Kinsella, J. P., Gien, J. & Frank, B. S. Failure to Normalize Biventricular Function Is Associated with Extracorporeal Membrane Oxygenation Use in Neonates with Congenital Diaphragmatic Hernia. *J. Pediatr.* **260**, 113490 (2023). 10.1016/j.jpeds.2023.113490 37201678

[44] Mashayekhi, B., Mohseni-Badalabadi, R., Hosseinsabet, A. & Ahmadian, T. Correlation between Heart rate recovery and Left Atrial phasic functions evaluated by 2D speckle-tracking Echocardiography after Acute Myocardial infarction. *BMC Cardiovasc. Disord.* **23**, 164 (2023). 10.1186/s12872-023-03194-y PMC10061796 36991359

