# Supplementary material for: Early left ventricular diastolic function and disease severity in congenital diaphragmatic hernia
Source: Pediatr Res. 2025 Nov 12;100(3):1158–65. doi: 10.1038/s41390-025-04560-6 (PMC13672081; doi:10.1038/s41390-025-04560-6)
Supplement: Supplementary file 1 [file 41390_2025_4560_MOESM1_ESM.pdf]

Table S1: Measures of LV diastolic function at <24h of age and at Pre-operative time points (n=27)

	**<24h of age**	**Pre-Operative**	**p**
**Age at echo. ,** hours median (range)	3 (1-24)/	168 (48-336)/	**-**
**Peak Mitral E** cm/sec, mean (SD)	43 (±11)	59 (±12)	**<0.001**
**Peak Mitral A** cm/sec, mean (SD)	48 (±13)	57 (±11)	0.06
**E/A ratio**, mean (SD)	0.93 (±0.3)	1.1 (±0.2)	**0.04**
**LV e’** cm/sec, mean (SD)	3.97 (±1.26)	4.1 (±1.3)	**<0.001**
**E: e’ ratio**, mean (SD)	11.6 (±4.3)	11.1 (±4.1)	0.65
**LASr** %, mean (SD)	22 (±8.7)	41.7 (±10.5)	**<0.001**
**LV LS** %, mean (SD)	-13.6 (±4.7)	-22.6 (±4.1)	**<0.001**
**RV LS** %, mean (SD)	-11.4 (±6.7)	-17.6 (±5.2)	**0.004**

E, peak early mitral diastolic inflow velocity; A, peak late mitral diastolic inflow velocity; E/A, ratio of peak mitral E and A diastolic velocities; LV e’, left ventricular early diastolic myocardial velocity; LV E:e’, ratio of mitral valve early diastolic filling velocity E and LV e’; LASr, left atrial reservoir strain; LV LS, peak left ventricular longitudinal strain; RV LS, peak right ventricular longitudinal strain.
